# The Effectiveness of Dry Needling in Patients with Hip or Knee Osteoarthritis: A Systematic Review and Meta-Analysis

**DOI:** 10.3390/life12101575

**Published:** 2022-10-11

**Authors:** Sandra Jiménez-del-Barrio, Ricardo Medrano-de-la-Fuente, Ignacio Hernando-Garijo, María Teresa Mingo-Gómez, Elena Estébanez-de-Miguel, Luis Ceballos-Laita

**Affiliations:** 1 Clinical Research in Health Sciences Group, Department of Surgery, Ophthalmology and Physiotherapy, University of Valladolid, Calle Universidad s/N, 42004 Soria, Spain; 2 Department of Physiatrist and Nursery, Faculty of Health Sciences, University of Zaragoza, Calle Domingo Miral s/N, 50009 Zaragoza, Spain

**Keywords:** dry needling, osteoarthritis, systematic review, meta-analysis

## Abstract

Background: Osteoarthritis is one of the most common degenerative joint diseases. The main symptoms of the osteoarthritis have been linked to the presence of myofascial trigger points in the soft tissues. Dry needing (DN) is the most investigated technique for the treatment of myofascial trigger points. Thus, the aim of this study was to evaluate the effectiveness of DN in pain and physical function in patients with osteoarthritis in the short-, medium- and long-term. Methods: PubMed, Cochrane Library, PEDro, Web of Science, and SCOPUS databases were searched in September 2022. Randomized controlled trials involving DN compared to non-pharmacological interventions, sham techniques or no additional treatment were selected. Quality of the studies was assessed with PEDro scale and risk of bias with Cochrane Collaboration tool. Meta-analyses were conducted using fixed or random effects models according to the Cochrane handbook for systematic reviews of interventions. Results: Seven studies were included in the meta-analysis involving 291 patients with osteoarthritis. The methodological quality of the included studies ranged from fair to high. DN showed significant improvements in pain intensity (SMD = −0.76; 95% CI: −1.24, −0.29; I^2^: 74%) and physical function (SMD = −0.98; 95% CI: −1.54, −0.42; I^2^: 75%) in the short-term. No differences were found in the medium- or long-term. The risk of bias, heterogeneity, and imprecision of the results downgraded the level of evidence to very low. Conclusions: Very low-quality evidence suggests a positive effect of DN for reducing pain intensity and improving physical function in the short term in patients with osteoarthritis. Further investigation is needed to determine a medium- and long-term effects.

## 1. Introduction

Osteoarthritis (OA) is a chronic condition characterized by degeneration of all of the tissues around the joint such as articular cartilage, synovial membrane, capsule and ligaments, and soft tissues, causing pain, stiffness, restricted range of motion, and physical function limitations [1]. The knee is the most prevalent joint affected by OA, followed by the hip and the thumb [2]. The prevalence of OA in Europe currently ranges from 2% to 17%, and is increasing in number and age, which means a high economic and social burden on worldwide population [2].

Pain is the symptom for which most patients seek medical attention but is not directly linked to radiographical findings [3]. Previous studies have suggested that soft tissue disfunctions may be related to the development and progression of OA [4,5,6,7,8,9]. Slemenda et al. [4] found that asymptomatic patients with OA presented muscle weakness but no muscle atrophy. Nguyen et al. [5] hypothesized that muscle weakness may be related to Myofascial Trigger Points (MTrPs). Patients with hip and knee OA have shown a higher prevalence of MTrPs than healthy adults [10], and an association between the presence of MTrPs in the soft tissues and osteoarthritic pain and physical function limitations has been found [11].

A MTrP is a hyperirritable nodule in a taut band of skeletal muscle. MTrPs may produce local and referred pain, limited range of motion, and muscle weakness. The MTrPs are classified as active or latent depending on whether they reproduce the patient’s pain or not [12]. Several treatment techniques have been described for the management of MTrPs and are commonly divided as non-invasive and invasive. Non-invasive techniques include stretching, friction massage, ischemic compression, or myofascial release among others. Dry needling (DN) is the most common invasive technique and consists of the insertion of a single-use acupuncture needle into the MTrP [13]. The Hong’s technique is the most investigated procedure and is based on eliciting local twitch responses after repeated needle insertion [14,15].

In recent years, several studies have analysed the effects of DN therapy in different musculoskeletal disorders [16,17,18]. Three systematic reviews and meta-analysis tried to address this topic [19,20,21]. El-Bachiri et al. [19] found no positive effects of DN in pain and physical function in patients with knee OA but only two studies were included. Ughreja et al. [21] and Lin et al. [20] found positive effects of different needling therapies in pain and physical function in the short-term in patients with knee OA. However, Ughreja et al. [21] analysed the effects of periosteal stimulation and intramuscular electrical stimulation, and Lin et al. [20] mainly included acupuncture interventions. Furthermore, these three studies included only patients with knee OA. Therefore, to the best of our knowledge, no study has assessed the effectiveness of DN therapy in pain and physical function in patients with OA in any joint.

Thus, the aim of the study was to investigate the effectiveness of DN therapy to improve pain and physical function in patients with OA in any joint in the short-, medium-, and long-term.

## 2. Materials and Methods

### 2.1. Study Design

The protocol of this systematic review and meta-analysis was registered in the International Prospective Register of Systematic Reviews (PROSPERO) with the registration number CRD42022359054. The study was carried out following the Preferred Reporting Items for Systematic Reviews and Meta-Analysis (PRISMA) statement and Cochrane Recommendations [22].

### 2.2. Search Strategy

The bibliographical search was conducted in PubMed (MEDLINE), Physiotherapy Evidence Database (PEDro), Cochrane Library, Web of Science (WOS), and SCOPUS from inception to 8 September 2022. The Population, Intervention, Comparison, and Outcome (PICO) framework was used to define the search strategy. Medical Subject Headings (MeSH) were used for the keywords: dry needling, osteoarthritis, hip osteoarthritis, knee osteoarthritis, and thumb osteoarthritis. All of the keywords used for the search strategy are described in Table 1. The search strategies used for each database are shown in Appendix A. SCOPUS database was included as a tool for searching grey literature, and a hand search of the reference list of the included studies was performed.

### 2.3. Eligibility Criteria and Study Selection

The included studies met the PICOs criteria: (1) patients clinically or radiographically diagnosed with OA following the American College of Rheumatology criteria [23] or the Kellgren-Lawrence criteria [24]; (2) DN therapy in the MTrPs of the soft tissues surrounding the affected joint in isolation or combined with other non-pharmacological techniques; (3) comparison with other non-pharmacological or non-surgical interventions, sham or simulated techniques, or no interventions; (4) outcomes consisted of pain intensity and/or physical function; (5) randomized controlled trials.

Studies were excluded if: (1) included patients without OA diagnosis or after total joint replacement; (2) included other types of needling interventions different from DN such as pharmacological injections, acupuncture, periosteal stimulation, or intramuscular electrical stimulation; (3) the intervention was performed in acupuncture points instead of MTrPs; (4) the outcome variables reported were not the outcomes of interest or were not measured using a valid and reliable instrument; (5) the design was not a randomized clinical trial or was a conference or congress abstract.

After searches were retrieved, references were exported to Mendeley desktop, and duplicates were removed. Two reviewers independently (LC and SJ) assessed the title and abstract of each reference to determine potential eligibility. The same independent reviewers assessed potential full texts. A third author (IH) resolved the discrepancies between the two reviewers.

### 2.4. Data Extraction

The two authors independently extracted the data from the identified studies using the standardized process adapted from the Cochrane Collaboration. Extracted information included: (1) characteristics of the study population; (2) aspects of the interventions applied in the experimental and control groups; (3) outcome measures; (4) results; and (5) follow-up period. Data were analysed using a qualitative synthesis and, whenever possible, using a quantitative synthesis (meta-analysis).

### 2.5. Risk of Bias and Quality of Evidence

Two reviewers assessed the quality of the studies using the PEDro scale and the Cochrane Risk of Bias tool. PEDro scale is an 11-items scale based on the Delphi list developed by Verhagen and Colleagues [25]. One item of the PEDro scale (eligibility criteria) is related to external validity and was not used to calculate the total score. A score of 7 or above was considered “high” quality, 5–6 was considered “fair” quality, and 4 or below was considered “poor quality” [25,26]. The Cochrane Risk of Bias tool determines the potential bias and the internal validity of the studies and classifies them as “low”, “unclear”, or “high” risk based on 7 criteria [27]. Both tools have shown to be reliable for evaluating the quality of the studies and assessing the risk of bias. The funnel plots are presented with a description of the risk of bias of each study assessed in the Appendix B.

The Grading of Recommendations Assessment, Development and Evaluation (GRADE) was used to develop a summary of the findings. This classification categorizes the evidence as “high”, “moderate”, “low”, or “very low” and allows us to discern the importance of the results. The quality of evidence for the meta-analysis was downgraded according to the presence of risk of bias, inconsistency of results, indirectness of evidence, and imprecision. To evaluate the risk of bias, the quality of evidence was downgraded by one level if more than 25% of the participants were from studies with poor or fair methodological quality (lack of allocation concealment, random allocation and/or sample size calculation, and participant, personnel and outcome assessors blinding) and was downgraded by two levels if was more than 50% of the participants. To evaluate the inconsistency of results, the quality of the evidence was downgraded by one level if significant heterogeneity was present by visual inspection or if the I^2^ value was greater than 50% and was downgraded by two levels if I^2^ value was greater than 75%. To evaluate the indirectness of evidence, the quality of the evidence was downgraded by one level if other populations, interventions, or comparators than considered in the objectives were included. To evaluate the imprecision, the quality of the evidence was downgraded by one level if the 95% Confidence Interval (95%CI) of the Standardized Mean Difference (SMD) was > 0.2 points, or by two levels if the difference was > 0.5 points. Moreover, one level was downgraded if fewer than 50 participants were included in the comparison, or two levels if fewer than 30 participants were included. GRADE table was performed using the GRADE Pro software, according to the data retrieved from the studies and the PEDro scale [27,28,29].

### 2.6. Data Synthesis and Analysis

The quantitative synthesis of the results was carried out according to the outcomes considered: pain intensity and physical function. When studies used different tools to assess the same outcome, the authors performed inverse variance methods.

Two different meta-analysis were performed for the results of pain and physical function. Mean and standard deviation (SD) on the post-intervention, and sample size from each group were extracted. SMD and 95% CI were calculated based on the post-intervention means and SDs.

Subgroup analysis of the studies were performed to compare DN therapy to other non-pharmacological or non-surgical interventions, sham or simulated techniques, or no interventions in the medium-term and the long term. Significance was set at a *p* value < 0.05.

Data were combined for meta-analysis using a minimum of two trials assessed as clinically homogeneous. Trials were considered clinically homogeneous if there was a common intervention and outcome. A fixed-effect meta-analysis was performed when each study estimated precisely the same quantity. Random-effect meta-analysis was performed when the combination of intervention effects could incorporate an assumption that the studies are not all estimating the same intervention effect [30]. Data on outcomes of interest were analysed by a researcher using RevMan 5.4 software.

## 3. Results

### 3.1. Literature Search and Screening

**Figure 1:**
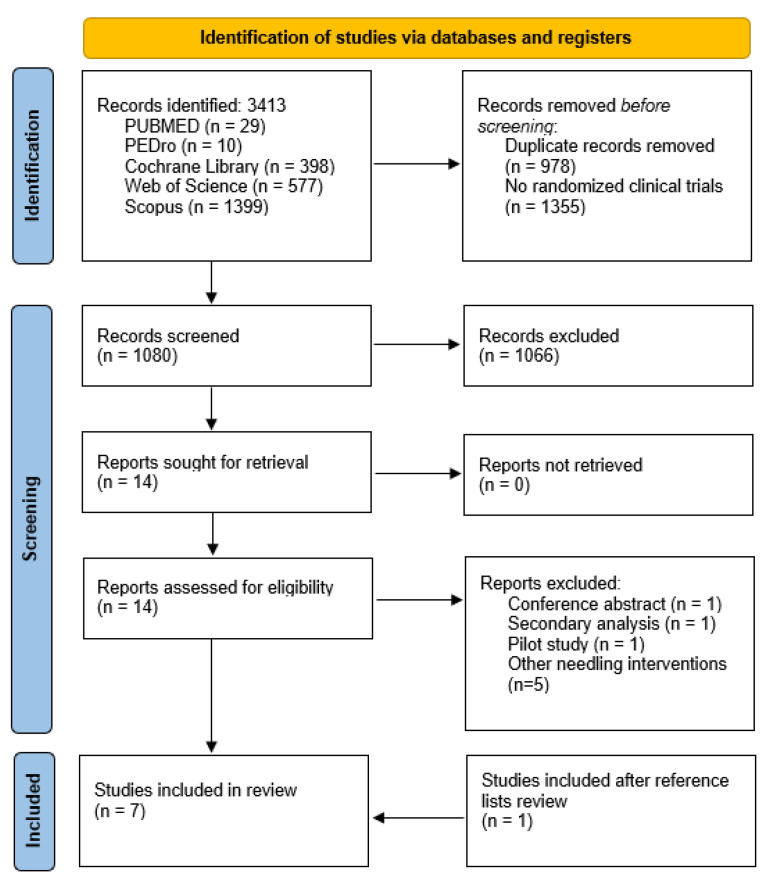
PRISMA flowchart of the study.

Seven studies were included in the qualitative and quantitative synthesis. Three studies were excluded after reading the full text, one was a pilot study [31], other was a conference abstract [32], and the other a secondary analysis of a randomized controlled trial [33]. The description of the selection process is shown in the PRISMA flowchart diagram (Figure 1). The agreement between reviewers was calculated by kappa with a value of 1.0.

### 3.2. Characteristics of the Eligible Studies

A total of 7 randomized controlled trials were included comprising 291 patients with hip or knee OA. The sample size ranged from 15 to 62 patients.

Three studies included patients with hip OA, and four studies included patients with knee OA. Six studies included patients with hip or knee OA clinically and radiographically diagnosed according to the American College of Rheumatology criteria and the Kellgren-Lawrence criteria, respectively [34,35,36,37,38,39]. Only one study included patients with knee OA diagnosed just following the Kellgren-Lawrence criteria [40]. The sociodemographic and clinical characteristics of the participants in each study are shown in Table 2.

The DN group in each trial consisted of DN therapy. Six studies applied DN in isolation [34,36,37,38,39,40], and one study applied DN plus exercise therapy [35]. Concerning the DN interventions, all of the included studies identified MTrPs according to the criteria described by Travell and Simons [12]. The targeted muscles and the needles used for each muscle varied across the studies. All of the studies performed rapid needle insertions to elicit local twitch responses [34,35,36,37,38,39,40]. The total number of local twitch responses was not registered in any study (Table 3).

The control group consisted of sham interventions, exercise therapy, self-stretching techniques, or no additional treatment. Five studies applied a sham intervention using a “sparrow pecking” technique [34,36,37,38,40], one study used no additional intervention [38], one study applied exercise therapy [35], and one study used a self-stretching protocol [39].

The number of sessions per week and the total number of sessions varied across the studies. The most common frequency was 1 session per week [34,35,36,37,38,39]. Only one study applied 3 treatment sessions in one week [40]. The total number of sessions ranged from 1 to 6 [34,35,36,37,38,39,40].

### 3.3. Outcome Measures

The outcomes considered in this meta-analysis were pain intensity and physical function. Seven studies assessed pain intensity. Six studies used the visual analogue scale (VAS) [34,35,36,37,38,40], and one study used the pain subscale of the Western Ontario and McMaster Universities Index of Osteoarthritis (WOMAC) questionnaire [39]. Physical function was measured in six studies. Four studies used the WOMAC questionnaire [34,35,38,39]. Two studies used the Self-Paced Walk Test (SPWT) [37,40].

All of the studies assessed the outcome variables at baseline and after intervention (short-term) [34,35,36,37,38,39,40]. Concerning the follow-up periods, 2 studies assessed the medium- and long-term results [34,35].

### 3.4. Study Quality and Risk of Bias

**Figure 2:**
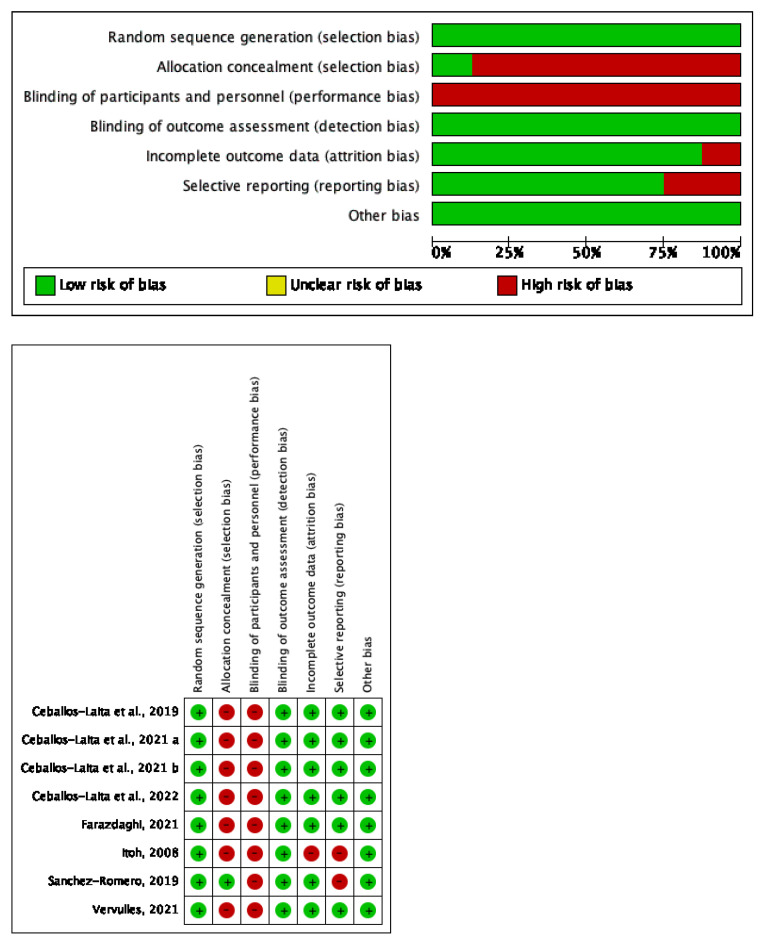
Risk of bias Cochrane tool [34,35,36,37,38,39,40].

Most of the randomized controlled trials included in this review described a high risk of selection and performance bias. All of the studies generated a random sequence and blinded the outcome examiners. The lack of therapist blinding is expected in conservative non-pharmacological interventions [41]. The Cochrane risk-of-bias tool results are shown in Figure 2.

According to the PEDro scale, one studies presented fair quality [34], and 6 studies presented high quality [35,36,37,38,39,40] (Table 4).

### 3.5. Synthesis of Results

#### 3.5.1. Pain Intensity

**Figure 3:**
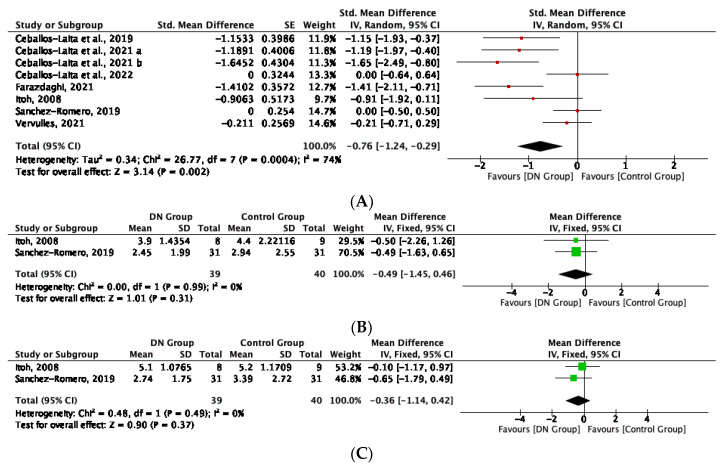

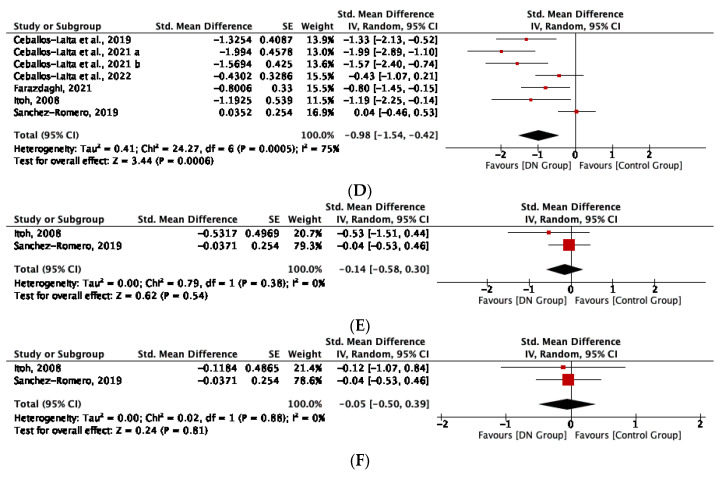
(**A**) Forest plot of pain intensity for DN therapy versus sham, exercise or no intervention in the short-term. (**B**) Forest plot of pain intensity for DN therapy versus sham or exercise intervention in the medium-term. (**C**) Forest plot of pain intensity for DN therapy versus sham or exercise intervention in the long-term. (**D**) Forest plot of physical function for DN therapy versus sham, exercise, or no intervention in the short-term. (**E**) Forest plot of physical function for DN therapy versus sham or exercise intervention in the medium-term. (**F**) Forest plot of physical function for DN therapy versus sham or exercise intervention in the long-term [34,35,36,37,38,39,40].

Pain intensity was measured in 7 studies in the short-term [34,35,36,37,38,39,40]. All of the studies were included in the quantitative synthesis, and meta-analysis showed that DN intervention produced significant improvement in pain intensity compared to sham techniques, exercise therapy or no intervention in the short-term (SMD = −0.76; 95% CI: −1.24, −0.29; I^2^: 74%) (Figure 3A).

Two studies measured pain intensity in the medium, and long-term [34,35]. All of the studies were included in the quantitative synthesis, and meta-analysis showed that DN interventions produced no significant improvements in pain intensity in the medium-term (Mean Difference (MD) = −0.49; 95% CI: −1.45, 0.46; I^2^: 0%) (SMD = −0.22; 95%IC: −0.66, 0.22; I^2^: 0%) (Figure 3B), or long-term (MD = −0.36; 95% CI: −1.14, 0.42; I^2^: 0%) (SMD = −0.24; 95%IC: −0.78, 0.22; I^2^: 0%) (Figure 3C).

#### 3.5.2. Physical Function

Physical function was measured in 6 studies in the short-term [34,35,37,38,39,40]. All of the studies were included in the quantitative synthesis, and meta-analysis showed that DN intervention produced significant improvement in physical function compared to sham techniques, exercise therapy or no intervention in the short-term (SMD = −0.98; 95% CI: −1.54, −0.42; I^2^: 75%) (Figure 3D).

Two studies measured physical function in the medium- and long-terms [34,35]. All of the studies were included in the quantitative synthesis, and meta-analysis showed that DN interventions produced no significant improvements in physical function in the medium-term (SMD = −0.14; 95% CI: −0.58, 0.30; I^2^: 0%) (Figure 3E), or long-term (SMD = −0.05; 95% CI: −0.50, 0.39; I^2^: 0%) (Figure 3F).

The overall quality of evidence according to GRADE was rated as very low for pain intensity and physical function (Appendix C).

#### 3.5.3. Adverse Events

Only Sanchez-Romero et al. [35] provided data about the adverse events. The study reported that 96.8% of the participants suffered post-needling soreness. It is also described that the participants commonly referred to hematoma and bleeding, but no serious adverse events were reported.

## 4. Discussion

The aim of this study was to investigate the effects of DN therapy for the management of pain and physical function in patients with OA. The present systematic review and meta-analysis found very-low quality evidence suggesting that DN therapy is more effective than sham techniques, exercise therapy or no intervention for reducing pain intensity and improving physical function in patients with hip or knee OA in the short-term. No serious adverse events were observed in the included studies. However, due to the risk of bias, inconsistency and imprecision, the level of evidence was downgraded to very low.

The methodological quality of the included randomized control trials ranged from fair to high on the PEDro scale. Common methodological flaws were lack of allocation concealment, blinding therapist, and intention-to-treat analysis. It is important to consider that the therapist blinding is not possible in non-pharmacological interventions [41]. Despite of the methodological quality of the included studies, the quality of the evidence was rated as very low. The quality of the evidence was downgraded because: more than 25% of the participants were from studies with lack of allocation concealment and the therapists were not blinded; the I^2^ value was higher than 75%; different comparators were included; and the 95%IC of the SMD was higher than 0.5 points.

Previous studies have suggested a positive effect of needling therapies for the treatment of knee OA [20,21]. However, these systematic reviews and meta-analysis did not include studies about DN. Our meta-analysis is the first to specifically investigate the effects of DN in patients with OA in any joint in the short-, medium- and long-term. This meta-analysis included 7 studies and indicated that the application of DN therapy in the MTrPs of the soft tissues in patients with hip or knee OA showed an immediate decrease in pain intensity and an immediate increase in physical function. The changes achieved were large in pain intensity (SMD = 0.76) and physical function (SMD = 0.98). Our results were higher to those obtained by El-Bachiri et al. [19], who found no benefits applying DN therapy in patients with knee OA. These differences could be because only two studies were included [34,35] and the interventions and comparisons were different between them. Itoh et al. [34] performed a randomized controlled trial comparing DN therapy to sham DN while Sanchez-Romero et al. [35] compared DN plus exercise therapy to exercise therapy in isolation. Therefore, each included study performed a different intervention and comparison, showing a high heterogeneity, which could condition the results.

Only two studies assessed the medium- and long-term effects, and the changes were not statistically significant. The results showed that DN therapy produced no benefits in pain intensity and physical function in patients with knee OA in the medium- or long-term follow-up. These results must be interpreted with caution due to the small numbers of studies that assessed follow-up periods.

Five studies applied DN therapy in isolation and was compared to a sham DN or a control group [34,36,37,38,40]. In the qualitative synthesis, 4 of the 5 studies reported positive effects in pain intensity and physical function. Vervullens et al. [36] was the only one that reported no statistically significant differences in pain intensity but only one single session of DN was applied. Sanchez-Romero et al. [35] compared DN therapy plus exercise to exercise in isolation, and Ceballos-Laita et al. [39] compared DN therapy to a self-stretching protocol. These two studies did not find better effects in pain intensity the group that received DN. The effects of DN in multimodal approaches are not well-described, so its combination with other techniques may conditioned the results. In this way, there is a need to know the effects of the DN therapy in combination to other interventions because of the multimodal approaches have more clinical applications.

The muscles treated with DN therapy varied across the studies. Iliopsoas, sartorious, quadriceps, tensor fasciae latae, gluteus medius, gluteus minimus, gluteus maximus, hamstrings, adductors, gastrocnemius, and popliteus muscles were the most treated. In this sense, there is a need to determine which muscles are more relevant for the treatment of each type of OA. Then, future DN protocols may include the most relevant muscles. In addition, it is important to consider its safety. The studies included in this systematic review and meta-analysis did not report any severe adverse event. However, Halle and Halle [42] described in detail possible adverse events that can occur depending on the treatment area. Most of them are minor adverse effects but must be known to reduce the risk.

This systematic review and meta-analysis has some limitations. Our search strategy may have been limited by the omission of other databases. The heterogeneity found in the type and duration of the therapies, complicates the interpretation of our results. Methodological limitations include the insufficient sample size that could overestimate the results, and the lack of follow-up measurements of the studies. Future studies should improve the quality of the studies to reduce the risk of bias, considering the allocation concealment and blinding of participants and researchers. The total number of sessions and the duration of the intervention should be described to allow for the replication and comparison of the study. Finally, the combination of therapies that produces the best effects should be investigated, as well as their dose.

## 5. Conclusions

This systematic review and meta-analysis found very low-quality evidence suggesting a positive effect of DN therapy in pain and physical function compared to sham, exercise, or control interventions in the short-term in patients with hip and knee OA. Further investigation is needed to determine the medium- and long-term effects and to determine the best multimodal intervention.

## Figures and Tables

**Table 1:** Keywords used for the search strategy.

Population	Intervention	Control	Outcomes
Osteoarthritis	Dry needling	-	symptom
Hip osteoarthritis	Trigger point acupuncture		pain
Knee osteoarthritis	Intramuscular stimulation		function
Thumb osteoarthritis			physical function
Spinal osteoarthritis			functional capacity

**Table 2:** Sociodemographic and clinical characteristics of the participants.

		Participants		Intervention			Outcome (Tool)	Main Results	Follow-Up
Author	N (Sex Ratio)	Mean Age (SD)	Diagnosis	DN Group	Control Group				
Itoh et al., 2008 [34]	15	DN: 74.20 (8.10) Control: 73.30 (6.50)	Knee OA	5 sessions (1 per week) (n = 8)	5 sessions of sham DN (1 per week) (n = 7)		-Pain (VAS) -Physical function (WOMAC)	↑ VAS and WOMAC in DN group vs. control group	↑ VAS and WOMAC in DN group vs. control group at 10 weeks of follow-up but not at 20 weeks of follow-up
Ceballos-Laita et al., 2019 [37]	30 (17M/13F)	DN: 55.5 (4.70)Control: 58.6 (6.60)	Hip OA	3 sessions (1 per week) (n = 15)	3 sessions of sham DN (1 per week) (n = 15)		-Pain (VAS) -Physical function (SPWT)	↑ VAS and SPWT in DN group vs. control group	No data
Sanchez-Romero et al., 2020 [35]	62 (18M/44F)	DN: 72.97 (6.29) Control: 71.65 (5.00)	Knee OA	6 sessions (1 per week) + Exercise therapy. (n =31)	Exercise therapy (n =31)		-Pain (VAS) -Physical function (WOMAC)	No between-groups differences	No between-groups differences at 6, 9 or 12 months of follow-up
Ceballos-Laita et al., 2021 [38]	45 (20M/25F)	DN: 57.53 (3.88) Control: 54.67 (4.48) Other: 58.20 (5.08)	Hip OA	3 sessions (1 per week) (n = 15)	3 sessions of sham DN (1 per week) (n = 15)	No additional intervention (n = 15)	-Pain (VAS) -Physical function (WOMAC PF)	↑ VAS, and WOMAC PF in DN group vs. control and sham groups	No data
Farazdaghi et al., 2021 [40]	40 (40F)	NR	Knee OA	3 sessions (3 per week) (n = 20)	3 sessions of sham DN in one week (n = 20)		-Pain (VAS) -Physical function (SPWT)	↑ VAS and SPWT in DN group vs. control group	No data
Vervullens et al., 2021 [36]	61 (27M/34F)	DN: 63.00 (10.00) Control: 66.00 (10.00)	Knee OA	1 session (n = 31)	1 session of sham DN (n = 30)		-Pain (VAS)	No between-groups differences	No data
Ceballos-Laita et al., 2022 [39]	38 (18M/20F)	DN: 53.60 (4.30) Control: 55.0 (4.10)	Hip OA	3 sessions (1 per week) (n = 19)	Self-stretching protocol for 3 weeks (n = 19)		-Pain (WOMAC P) -Physical function (WOMAC PF)	No between-groups differences	No data

M: male; F: female; SD: standard deviation; DN: dry needling; OA: osteoarthritis; VAS: visual analogue scale; WOMAC: Western Ontario and McMaster Universities Index of Osteoarthritis; WOMAC-P: Western Ontario and McMaster Universities Index of Osteoarthritis Pain subscale; WOMAC-PF: Western Ontario and McMaster Universities Index of Osteoarthritis Physical function subscale; SPWT: self-paced walk test.

**Table 3:** DN therapies of the included studies.

Study	MtrP Criteria	Muscles Treated	Gauge (mm)	LTR
Itoh et al., 2008 [34]	YES	quadriceps, iliopsoas, sartorius, adductors, popliteus, gluteus minimus and hamstrings	0.2 × 50	YES
Ceballos-Laita et al., 2019 [37]	YES	iliopsoas, rectus femoris, tensor fasciae latae, gluteus medius and minimus	0.25 × 50	YES
Sanchez-Romero et al., 2020 [35]	YES	NR	0.3 × 40 0.3 × 60 0.3 × 75	YES
Ceballos-Laita et al., 2021 [38]	YES	iliopsoas, rectus femoris, tensor fasciae latae, gluteus medius and minimus	0.25 × 50	YES
Farazdaghi et al., 2021 [40]	YES	hip adductors, abductors, flexors and extensors, and knee flexors and extensors	0.24 × 40	YES
Vervullens et al., 2021 [36]	YES	gastrocnemius, vastus medialis, vastus lateralis, rectus femoris, biceps femoris, semitendinosus, semimembranosus, adductor longus, adductor brevis	0.3 × 40 0.3 × 70	YES
Ceballos-Laita et al., 2022 [39]	YES	iliopsoas, rectus femoris, tensor fasciae latae, gluteus medius and minimus	0.25 × 50	YES

MTrP: myofascial trigger point; LTR: local twitch response; NR: no reported.

**Table 4:** PEDro scale scores.

Study	Items											Total Score
	1	2	3	4	5	6	7	8	9	10	11	
Itoh et al., 2008 [34]	Y	Y	N	Y	Y	N	Y	N	N	Y	Y	6/10
Ceballos-Laita et al., 2019 [37]	Y	Y	N	Y	Y	N	Y	Y	N	Y	Y	7/10
Sanchez-Romero et al., 2020 [35]	Y	Y	Y	Y	Y	N	Y	Y	N	Y	Y	8/10
Ceballos-Laita et al., 2021 [38]	Y	Y	N	Y	Y	Y	N	Y	N	Y	Y	7/10
Farazdaghi et al., 2021 [40]	Y	Y	N	Y	Y	N	Y	Y	Y	Y	Y	8/10
Vervullens et al., 2021 [36]	Y	Y	N	Y	N	N	Y	Y	Y	Y	Y	7/10
Ceballos-Laita et al., 2022 [39]	Y	Y	N	Y	Y	N	Y	Y	N	Y	Y	7/10

Y: yes; N: no. Eligibility criteria were specified. Subjects were randomly allocated. Allocation was concealed. The groups were similar at baseline. There was blinding of all subjects. There was blinding of all therapists who administered the therapy. There was blinding of all assessors who measured at least one key outcome. Measures of at least one key outcome were obtained from more than 85% of the subjects initially allocated to groups. All subjects for whom outcome measures were available received the treatment or control condition as allocated or, where this was not the case, data for at least one key outcome was analyzed by intention to treat. The results of between-group statistical comparisons are reported for at least one key outcome. The study provides both point measures and measures of variability for at least one key outcome.

**Figure A1:**
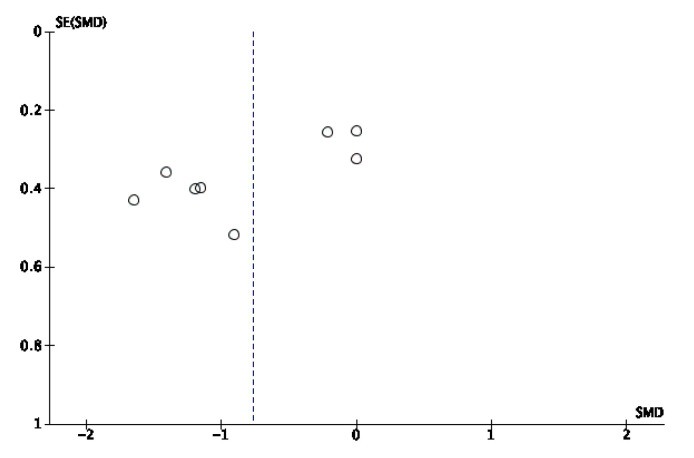
Funnel plot pain intensity in the short-term.

**Figure A2:**
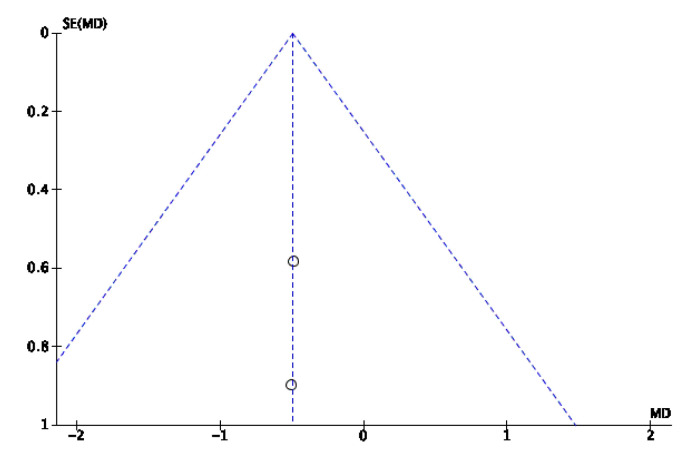
Funnel plot pain intensity in the medium-term.

**Figure A3:**
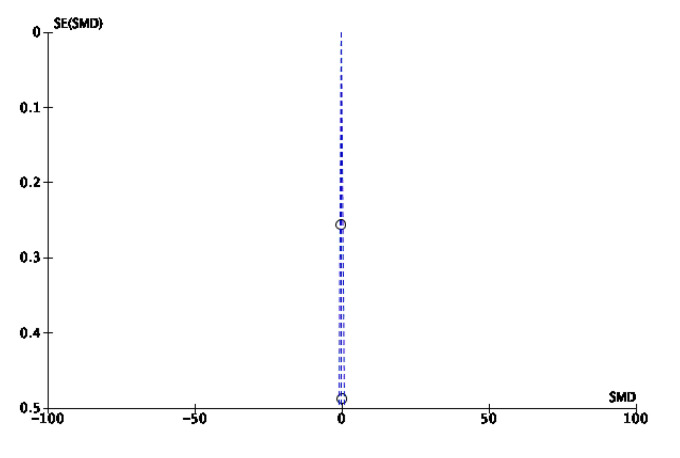
Funnel plot pain intensity in the long-term.

**Figure A4:**
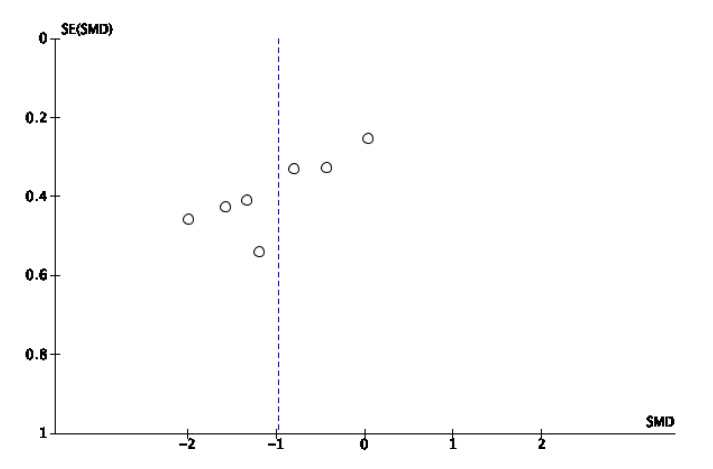
Funnel plot physical function in the short-term.

**Figure A5:**
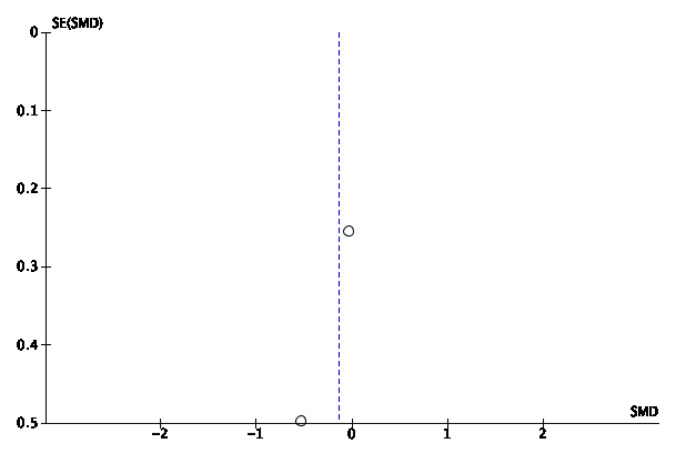
Funnel plot physical function in the medium-term.

**Figure A6:**
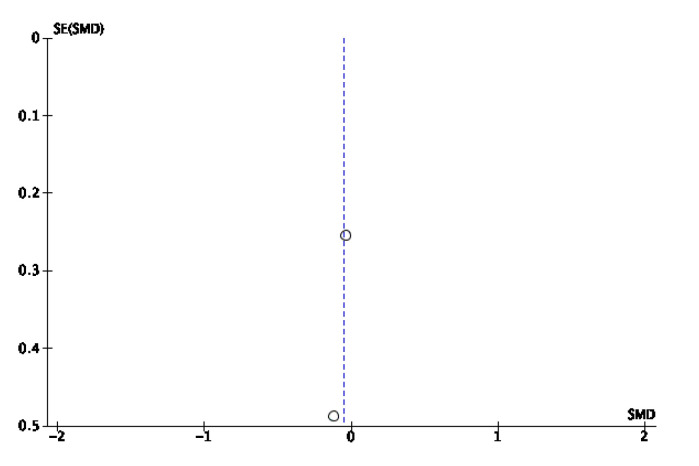
Funnel plot physical function in the long-term.

## Data Availability

The anonymized data are available from the author upon reasonable request.

## Appendix A. Search Strategy

**#1** (osteoarthritis OR “spine osteoarthritis” OR “hip osteoarthritis” OR “knee osteoarthritis” OR “thumb osteoarthritis”)

**#2** (Dry needling OR acupuncture OR trigger point acupuncture OR intramuscular stimulation)

**#3** (symptom* pain OR functi* OR function OR physical function OR functional capacity)

**PUBMED**

Formula: #1 AND #2 AND #3

Results: 29

Data: 11 September 11 2022

**PEDro**

Formula: #1 AND #2

Results: 10

Data: 11 September 2022

**COCHRANE Library**

Formula: #1 AND #2 AND #3

Results: 398

Data: 11 September 2022

**Web of Science**

Formula: #1 AND #2 AND #3

Results: 577

Data: 11 September 2022

**SCOPUS**

Formula: #1 AND #2 AND #3

Results: 1399

Data: 11 September 2022

## Appendix C. GRADE Summary

**Table A1:** GRADE Summary of evidence of the results according to their certainly and their importance using the GRADE tool.

Assessment Cernainly							No. de Pacientes		Effect		Certainly	Importance
No. of Studies	Study Design	Risk of Bias	Inconsistency	Indirectly Evidence	Imprecision	Otrhers	[Intervention Group ]	[Comparation Group]	Relative(95% CI)	Absolute(95% CI)		
Pain intensity short-term												
7	RCTs	Serious ^a^	Serious ^b^	Not serious	Serious ^d^	None	131	115	-	SMD **0.75 less** (1.27 less to 0.23 less)	⨁◯◯◯Very Low	Critical
Function short term												
6	RCTs	Serious ^a^	Serious ^b^	Not serious	Serious ^d^	None	100	30	-	SMD **0.96 less** (1.58 less to 0.34 less)	⨁◯◯◯Very Low	Critical
Pain intensity medium term												
2	RCTs	Serious ^a^	Serious ^c^	Not serious	Serious ^e^	None	39	38	-	SMD **0.49 less** (1.45 less to 0.46 higher)	⨁◯◯◯Very Low	Critical
Pain intensity long term												
2	RCTs	Serious ^a^	Serious ^c^	Not serious	Serious ^e^	None	39	38	-	SMD **0.24 less** (0.68 less to 0.2 higher)	⨁◯◯◯Very Low	Critical
Function medium term												
2	RCTs	Serious ^a^	Serious ^c^	Not serious	Serious ^e^	None	39	38	-	SMD **0.14 less** (0.58 less to 0.3 higher)	⨁◯◯◯Very Low	Critical
Function long term												
2	RCTs	Serious ^a^	Serious ^c^	Not serious	Serious ^e^	None	39	38	-	SMD **0.05 less** (0.5 less to 0.39 higher)	⨁◯◯◯Very Low	Critical

CI: Confidence interval; SMD: Standarized mean difference; RCTs: Randomized controlled trials. EXPLANATIONS: ^a^. More than 25% of the participants were from studies with poor or fair methodological quality, considering this aspects: Lack of allocation concealment, random allocation and/or sample size calculation, participant and personnel blinding, blinding of outcome assessors. ^b^. Significant heterogeneity of the studies or if the I^2^ value was greater than 50%. ^c^. Significant heterogeneity of the studies (different interventions in the control and intervention groups). ^d^. The 95% CI of the SMD was <0.2 points. ^e^. Fewer than 100 participants were included. High: We are very confident that the true effect is close to the estimate of the effect. Moderate: We are moderately confidence in the effect estimate. The true effect is close to the estimate of the effect, but the result can be different. Low: Confidence in the effect estimate is limited, the true effect can be substantially different from the estimate of the effect. Very Low: There is little confidence in the effect estimate, the true effect is likely to be substantially different from the estimate effect.

## References

[1] Bennell K. (2013). Physiotherapy management of hip osteoarthritis. J. Physiother..

[2] Guillemin F., Rat A., Mazieres B., Pouchot J., Fautrel B., Euller-Ziegler L., Fardellone P., Morvan J., Roux C., Verrouil E. (2011). Prevalence of symptomatic hip and knee osteoarthritis: A two-phase population-based survey. Osteoarthr. Cartil..

[3] Pereira D., Severo M., Santos R.A., Barros H., Branco J., Lucas R., Costa L., Ramos E. (2015). Knee and hip radiographic osteoarthritis features: Differences on pain, function and quality of life. Clin. Rheumatol..

[4] Slemenda C., Brandt K.D., Heilman D.K., Mazzuca S., Braunstein E.M., Katz B.P., Wolinsky F. (1997). Quadriceps Weakness and Osteoarthritis of the Knee. Ann. Intern. Med..

[5] Nguyen B.M. (2013). Myofascial trigger point, falls in the elderly, idiopathic knee pain and osteoarthritis: An alternative concept. Med. Hypotheses.

[6] Becker R., Berth A., Nehring M., Awiszus F. (2004). Neuromuscular quadriceps dysfunction prior to osteoarthritis of the knee. J. Orthop. Res..

[7] Roos E.M., Herzog W., Block J., Bennell K. (2010). Muscle weakness, afferent sensory dysfunction and exercise in knee osteoarthritis. Nat. Rev. Rheumatol..

[8] Segal N., Glass N. (2011). Is Quadriceps Muscle Weakness a Risk Factor for Incident or Progressive Knee Osteoarthritis?. Phys. Sportsmed..

[9] Herzog W., Longino D., Clark A. (2003). The role of muscles in joint adaptation and degeneration. Langenbeck’s Arch. Surg..

[10] Dor A., Kalichman L. (2017). A myofascial component of pain in knee osteoarthritis. J. Bodyw. Mov. Ther..

[11] Sánchez Romero E.A., Fernández Carnero J., Villafañe J.H., Calvo-Lobo C., Ochoa Sáez V., Burgos Caballero V., Pecos Martín D. (2020). Prevalence of myofascial trigger points in patients with mild to moderate painful knee osteoarthritis: A secondary analysis. J. Clin. Med..

[12] Simons D., Travell J.G., Simons L. (2007). Myofascial Pain and Dysfunction: The Trigger Point Manual.

[13] Dommerholt J., Mayoral del Moral O., Gröbli C. (2006). Trigger Point Dry Needling. J. Man. Manip. Ther..

[14] Hong C.-Z., Torigoe Y. (1994). Electrophysiological Characteristics of Localized Twitch Responses in Responsive Taut Bands of Rabbit Skeletal Muscle Fibers. J. Musculoskelet. Pain.

[15] Hong C.-Z., Torigoe Y., Yu J. (1995). The Localized Twitch Responses in Responsive Taut Bands of Rabbit Skeletal Muscle Fibers Are Related to the Reflexes at Spinal Cord Level. J. Musculoskelet. Pain.

[16] Llurda-Almuzara L., Labata-Lezaun N., Meca-Rivera T., Navarro-Santana M.J., Cleland J.A., Fernández-De-Las-Peñas C., Pérez-Bellmunt A. (2021). Is Dry Needling Effective for the Management of Plantar Heel Pain or Plantar Fasciitis? An Updated Systematic Review and Meta-Analysis. Pain Med..

[17] Pourahmadi M., Dommerholt J., Fernández-De-Las-Peñas C., Koes B.W., Mohseni-Bandpei M.A., Mansournia M.A., Delavari S., Keshtkar A., Bahramian M. (2021). Dry Needling for the Treatment of Tension-Type, Cervicogenic, or Migraine Headaches: A Systematic Review and Meta-Analysis. Phys. Ther..

[18] Sánchez-Infante J., Navarro-Santana M.J., Bravo-Sánchez A., Jiménez-Diaz F., Abián-Vicén J. (2021). Is dry needling applied by physical therapists effective for pain in musculoskeletal conditions? a systematic review and meta-analysis. Phys Ther..

[19] Rahou-el-bachiri Y., Navarro-santana M.J., Guido F.G., Cleland J.A., Ibai L. (2020). Effects of Trigger Point Dry Needling for the Management of Knee Pain Syndromes: A Systematic Review and Meta-Analysis. J. Clin. Med..

[20] Lin X., Li F., Lu H., Zhu M., Peng T.Z., Albadrany Y. (2022). Acupuncturing of myofascial pain trigger points for the treatment of knee osteoarthritis: A systematic review and meta-analysis. Medicine.

[21] Ughreja R.A., Prem V. (2021). Effectiveness of dry needling techniques in patients with knee osteoarthritis: A systematic review and meta-analysis. J. Bodyw. Mov. Ther..

[22] Page M.J., McKenzie J.E., Bossuyt P.M., Boutron I., Hoffmann T.C., Mulrow C.D., Moher D. (2021). The PRISMA 2020 statement: An updated guideline for reporting systematic reviews. BMJ.

[23] Altman R., Alarcon G., Appelrouth D., Bloch D., Borenstein D., Brandt K., Wolfe F. (1991). The American College of Rheumatology criteria for the classifi-cation and reporting of osteoarthritis of the hip. Arthritis Rheum..

[24] Kellgren J.H., Lawrence J.S. (1957). Radiological Assessment of Osteo-Arthrosis. Ann. Rheum. Dis..

[25] Verhagen A.P., De Vet H.C., De Bie R.A., Kessels A.G., Boers M., Bouter L.M., Knipschild P.G. (1998). The Delphi list: A criteria list for quality assessment of randomized clinical trials for conducting systematic reviews developed by Delphi consensus. J. Clin. Epidemiol..

[26] Young J.L., Rhon D., de Zoete R.M., Cleland J.A., Snodgrass S.J. (2017). The influence of dosing on effect size of exercise therapy for musculoskeletal foot and ankle disorders: A systematic review. Braz. J. Phys. Ther..

[27] Higgins J.P., Altman D.G., Gøtzsche P.C., Jüni P., Moher D., Oxman A.D., Sterne J.A. (2011). The Cochrane Collaboration’s tool for assessing risk of bias in randomised trials. BMJ.

[28] Xie C.X., Machado G.C. (2020). Clinimetrics: Grading of Recommendations, Assessment, Development and Evaluation (GRADE). J. Physiother..

[29] Cohen J. (1988). Statistical Power Analysis for the Behavioral Cciences.

[30] Higgins J., Thomas J. (2019). Cochrane Handbook for Systematic Reviews of Interventions.

[31] Sánchez-Romero E.A., Pecos-Martín D., Calvo-Lobo C., Ochoa-Sáez V., Burgos-Caballero V., Fernández-Carnero J. (2018). Effects of dry needling in an exercise program for older adults with knee osteoarthritis. Medicine.

[32] Pang J.C.Y., Fu A.S.N., Lam S.K.H., Fu A.C.L. (2022). Effectiveness of ultrasound-guided dry needling in physiotherapy management of knee osteoarthri-tis: A randomized, double-blinded and controlled study. Physiotherapy.

[33] Ceballos-Laita L., Jiménez-Del-Barrio S., Marín-Zurdo J., Moreno-Calvo A., Bone J.M., Albarova-Corral M.I., Estébanez-De-Miguel E. (2020). Effects of dry needling on pain, pressure pain threshold and psychological distress in patients with mild to moderate hip osteoarthritis: Secondary analysis of a randomized controlled trial. Complement. Ther. Med..

[34] Itoh K., Hirota S., Katsumi Y., Ochi H., Kitakoji H. (2008). Trigger point acupuncture for treatmente of knee osteoarthritis. A preliminary RCT for a pragmatic trial. Acupunct Med..

[35] Sánchez Romero E.A., Fernández-Carnero J., Calvo-Lobo C., Ochoa Sáez V., Burgos Caballero V., Pecos-Martín D. (2020). Is a Combination of Exercise and Dry Needling Effective for Knee OA?. Pain Med..

[36] Vervullens S., Meert L., Baert I., Delrue N., Heusdens C.H.W., Hallemans A., Van Criekinge T., Smeets R.J.E.M., De Meulemeester K. (2021). The effect of one dry needling session on pain, central pain processing, muscle co-contraction and gait characteristics in patients with knee osteoarthritis: A randomized controlled trial. Scand. J. Pain.

[37] Ceballos-Laita L., Jiménez-Del-Barrio S., Marín-Zurdo J., Moreno-Calvo A., Bone J.M., Albarova-Corral M.I., Estébanez-De-Miguel E. (2019). Effects of dry needling in HIP muscles in patients with HIP osteoarthritis: A randomized controlled trial. Musculoskelet. Sci. Pract..

[38] Ceballos-Laita L., Jiménez-del-Barrio S., Marín-Zurdo J., Moreno-Calvo A., Marín-Boné J., Albarova-Corral M.I., Estébanez-de-Miguel E. (2021). Effectiveness of Dry Needling Therapy on Pain, Hip Muscle Strength and Physical Function in Patients with Hip Osteoarthritis: A Randomized Controlled Trial. Arch. Phys. Med. Rehabil..

[39] Ceballos-Laita L., Jiménez-Del-Barrio S., Marín-Zurdo J., Moreno-Calvo A., Marín-Boné J., Albarova-Corral M.I., Estébanez-De-Miguel E. (2022). Comparison of dry needling and self-stretching in muscle extensibility, pain, stiffness, and physical function in hip osteoarthritis: A randomized controlled trial. Complement. Ther. Clin. Pract..

[40] Farazdaghi M.R., Yoosefinejad A.K., Abdollahian N., Rahimi M., Motealleh A. (2021). Dry needling trigger points around knee and hip joints improves function in patients with mild to moderate knee osteoarthritis. J. Bodyw. Mov. Ther..

[41] Kamper S.J. (2018). Blinding: Linking Evidence to Practice. J. Orthop. Sports Phys. Ther..

[42] Halle J.S., Halle R.J. (2016). Pertinent dry needling considerations for minimizing adverse effects—Part one. Int. J. Sports Phys. Ther..

